# Open-access fNIRS dataset for motor imagery of lower-limb knee and ankle joint tasks

**DOI:** 10.3389/frobt.2025.1695169

**Published:** 2025-12-05

**Authors:** Haroon Khan, Hammad Nazeer, Hamza Shabbir Minhas, Noman Naseer, Peyman Mirtaheri

**Affiliations:** 1 Department of Mechanical, Electronic and Chemical Engineering, OsloMet – Oslo Metropolitan University, Oslo, Norway; 2 Department of Mechatronics and Biomedical Engineering, Air University Islamabad, Islamabad, Pakistan; 3 Human-Centred Technology Research Centre, Faculty of Science and Technology, University of Canberra, Canberra, ACT, Australia

**Keywords:** brain-computer interface, functional near-infrared spectroscopy, open access, motor imagery, dataset

## 1 Introduction

Brain-Computer Interface (BCI) is an advanced system that enables direct communication between the human brain and external devices, bypassing the need for muscle activity. BCI can translate brain signals into commands to control assistive technologies, offering support for individuals with severe motor impairments, such as those caused by stroke or spinal cord injury. BCI hold applications in healthcare, especially for restoring movement and interaction in patients with limited voluntary motor control. By leveraging neural activity, BCIs open pathways for motor rehabilitation, assistive device control, and even cognitive assessment, enhancing quality of life and promoting greater independence.

A widely used control strategy in BCI research is motor imagery (MI), which involves mentally simulating specific physical movements without any actual muscle activation. MI is considered a cognitively driven process that mimics the planning stages of real movements and activates neural networks involved in motor execution. This includes regions such as the primary motor cortex, supplementary motor area (SMA), and premotor cortex, supporting the “functional equivalence” hypothesis (Naseer and Hong, 2015). Because MI can be performed without physical movement, it is especially suitable for patients who are paralyzed, amputated, or recovering from neurological trauma, making it highly relevant for neurorehabilitation and assistive technology development.

Over the past decades, a variety of neuroimaging modalities have been employed to capture the neural correlates of MI for BCI applications. These include invasive techniques like intracranial EEG (iEEG) and non-invasive modalities such as electroencephalography (EEG), magnetoencephalography (MEG), functional magnetic resonance imaging (fMRI), and functional near-infrared spectroscopy (fNIRS) (Cutini and Brigadoi, 2014). EEG is widely favoured for its high temporal resolution and affordability; however, it is highly susceptible to electrical interference and motion artefacts. On the other hand, fMRI offers excellent spatial resolution but is often limited by its high cost, immobility, and operational complexity. These limitations restrict their practical use, especially in real-world or bedside BCI applications.

Among non-invasive techniques, fNIRS has emerged as a promising neuroimaging modality for BCI systems. fNIRS uses near-infrared light to measure cerebral hemodynamic responses, specifically tracking changes in oxygenated (ΔHbO) and deoxygenated hemoglobin (ΔHbR) concentrations in the cortex (Khan et al., 2020). When a brain region becomes active, local blood flow increases, leading to a rise in ΔHbO and a decrease in ΔHbR. fNIRS offers a balance between spatial and temporal resolution, is portable, non-invasive, relatively affordable, and less sensitive to movement artifacts compared to EEG or fMRI (Nazeer et al., 2020a). Its quiet, mobile nature also makes it more comfortable for users and suitable for long-duration BCI sessions. These advantages make fNIRS a particularly suitable tool for developing MI-based BCI systems aimed at real-world rehabilitation scenarios.

While MI has been extensively studied for upper limb movements, there is increasing interest in lower limb motor imagery tasks due to their clinical importance. Lower limb movements such as ankle dorsiflexion/plantarflexion and knee flexion/extension are fundamental to essential motor functions like walking, balance, and postural control (Brockett and Chapman, 2016). Understanding how these movements are represented in the brain during MI can help in developing effective rehabilitation protocols and control strategies for assistive devices. MI of lower limb tasks engages similar motor areas to actual movement, supporting its use in BCIs that aim to stimulate recovery or control robotic assistance.

Datasets focusing on lower limb MI are essential for advancing research in rehabilitation and assistive robotics. They provide the foundation for developing and training machine learning models that can accurately decode movement intent from brain signals. Several studies have shown that MI of knee or ankle movements can generate meaningful patterns suitable for decoding and can be used to control prosthetic limbs or lower limb exoskeletons (Colucci et al., 2022). For instance, joint EEG-fNIRS studies have demonstrated the effectiveness of decoding ankle dorsiflexion and its relevance to balance recovery in stroke patients (Liang et al., 2022). Similarly, several studies has been conducted to improve classification accuracy of fNIRS (Li et al., 2024; Li and Li, 2025; Li and Sun, 2025) and hybrid EEG-fNIRS signals (Xu et al., 2023; Wang et al., 2025) for motor imagery/execution tasks. Such insights are crucial for improving the responsiveness and reliability of BCI-driven assistive devices.

To support this line of research, we introduce a novel fNIRS dataset focused on lower limb MI tasks, specifically ankle dorsiflexion/plantarflexion and knee flexion/extension. The dataset is designed to facilitate the development of BCI systems intended for controlling lower limb exoskeletons in rehabilitation applications (Minhas et al., 2024a). By capturing the hemodynamic responses associated with these specific MI tasks, the dataset enables researchers to explore neural patterns linked to lower limb motor control and develop machine learning models for real-time BCI applications. The unique focus on lower limb motor tasks, combined with the use of fNIRS, distinguishes this dataset and highlights its potential for impactful contributions to neurorehabilitation, exoskeleton design, and assistive technology research.

## 2 Methodology

This section details the study’s methodology, including participant selection, data acquisition procedures, and the experimental paradigm for MI tasks.

### 2.1 Subjects

The study included 21 healthy participants (12 males and 9 females) with a mean age of 24 ± 2.1 years, along with one male participant (subject 22) who had a right above-knee ultra-short stump amputation. Ethical approval for the study was granted by the Ethical Committee at Air University (Approval No. AU/EA/2021/03/003). A pre-screening questionnaire was used to confirm that none of the participants had cardiovascular or neurological conditions, as such conditions could affect blood flow regulation or neural activation patterns (Pelicioni et al., 2019). Participants were instructed to avoid smoking and caffeine for at least 3 hours prior to the experiment (Sargent et al., 2020). The study adhered to the ethical principles of the Declaration of Helsinki (World Medical Association, 2013). All participants provided written informed consent after receiving a full explanation of the procedure and demographic details are shown in Table 1.

**TABLE 1:** Demographic details of all participants.

Participants demographic data						
S.No	Gender	Age	Education	Dominance	Neurological disorder	Psychiatric disorder
1	M	22	Undergraduate	Right	No	No
2	M	21	Undergraduate	Right	No	No
3	M	27	Graduate	Right	No	No
4	F	23	Graduate	Right	No	No
5	M	23	Graduate	Right	No	No
6	M	28	DAE	Right	No	No
7	M	34	Undergraduate	Right	No	No
8	M	26	Graduate	Right	No	No
9	F	21	Undergraduate	Right	No	No
10	F	21	Undergraduate	Right	No	No
11	F	18	Undergraduate	Right	No	No
12	M	21	Undergraduate	Right	No	No
13	F	20	Undergraduate	Right	No	No
14	M	18	Undergraduate	Right	No	No
15	F	27	Undergraduate	Right	No	No
16	F	21	Undergraduate	Right	No	No
17	F	19	Undergraduate	Right	No	No
18	F	18	Undergraduate	Right	No	No
19	M	23	Graduate	Right	No	No
20	M	24	Graduate	Right	No	No
21	M	21	Undergraduate	Right	No	No
22	M	28	Graduate	Right	No	No

### 2.2 Data acquisition

Eight sources and eight detectors were used to record fNIRS signals from the motor cortex, yielding twenty physiological channels. Optode placement followed the internationally recognized EEG 10–20 system (Homan et al., 1987), which ensures standardized and accurate localization of brain regions in neurophysiological research. A fixed source-detector distance of 3 cm was maintained using optode holders, as this spacing is optimal for capturing neural activity from the motor cortex, (Gagnon et al., 2014). Brain signals were recorded using the NIRSport2 system (NIRx Medical Technologies, Germany) using 760 nm and 850 nm wavelengths at a sampling rate of 10.1725 Hz. Data collection was managed using Aurora fNIRS software (NIRx Medical Technologies, Germany), while PsychoPy was used to design the experiment, send triggers, and run the task.

To ensure signal accuracy, secure attachment of optodes to the scalp was prioritized, particularly in participants with thick hair, which can obstruct optical signals (Kwasa et al., 2023). This was achieved using appropriately sized elastic caps equipped with spring-loaded grommets and holders for consistent scalp contact. Real-time signal monitoring via Aurora facilitated the identification and correction of poor contact points. Participants were also advised to wash their hair prior to the session to minimize interference from oil and dandruff.

### 2.3 Experimental paradigm

**FIGURE 1:**
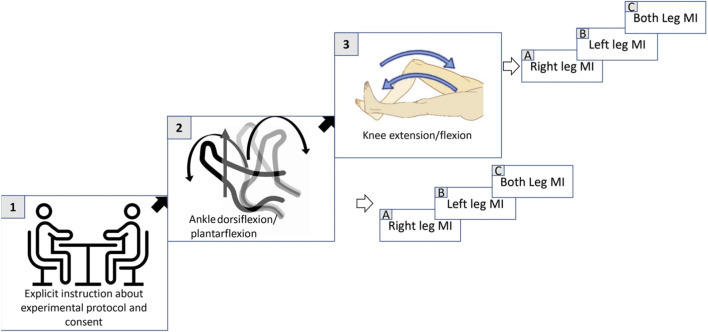
Overview of the experimental protocol. Participants received instructions and gave consent **(1)**, followed by MI tasks of the ankle **(2)** and knee **(3)**.

In this study, MI tasks included dorsiflexion and plantarflexion of the ankle joint, as well as flexion and extension of the knee joint. These tasks were performed sequentially using the right leg, left leg, and both legs. Participants were positioned in Fowler’s position (a 45-degree recline), which was selected to enhance cerebral blood flow (Kubota et al., 2017). The experiment took place in a quiet environment with reduced ambient light to minimize noise and optical interference (Solovey et al., 2009), thereby improving data accuracy by reducing artifacts. To differentiate between MI and ME, inertial measurement unit (IMU) sensors were secured to the participants’ ankle joints using adjustable straps. These sensors were placed for monitoring purpose and helped detect any unintended physical movements during the tasks (Mizuguchi et al., 2012). Participants were seated in front of a computer screen placed approximately 2 m away. Prior to the experiment, they were thoroughly briefed on the procedure and instructed to avoid any unnecessary movements (Shin et al., 2017). Trials were discarded if any physical movement was detected during the task. Visual cues were presented on the screen to guide participants throughout the experiment. Figure 1 shows the overall experimental protocol.

**FIGURE 2:**
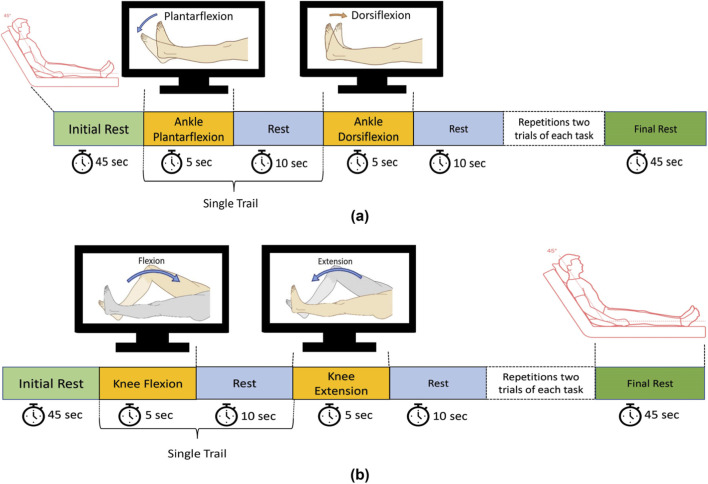
**(a)** Schematic of the experimental paradigm for ankle dorsiflexion and plantarflexion tasks; **(b)** Schematic of the experimental paradigm for knee flexion and extension tasks. Reprinted with permission from Minhas et al. (2024a), Copyright © 2024, IEEE.

The task began with a 45-s rest period to allow the hemodynamic response to stabilize. During this time, participants were instructed to remain still and avoid any voluntary movements. Following the initial rest, participants performed an MI task involving ankle plantarflexion, guided by on-screen instructions for a duration of 5 s. This was followed by a 10-s rest period to allow the hemodynamic signals to return to baseline (Nazeer et al., 2020b). Subsequently, participants engaged in an MI task involving ankle dorsiflexion for 5 s. This cycle of MI tasks and rest was repeated for three trials, followed by a final 45-s rest period to return to baseline. The same protocol was repeated for knee joint movements, specifically flexion and extension, following the same sequence and timing as used for ankle tasks. Figures 2A,B illustrate the experimental paradigms for ankle and knee movements, respectively.

### 2.4 Data preprocessing

Data preprocessing was carried out using Satori 2.0 (NIRx Medical Technologies, Germany). fNIRS signals are often affected by physiological artifacts such as respiration, cardiac pulsation, Mayer waves, and motion-related noise (Hakimi et al., 2023). To reduce artifacts, a fourth-order Butterworth bandpass filter was applied with a frequency range of 0.01–0.3 Hz (Minhas et al., 2024b). Z-transform normalization was also applied to standardize the data, making it easier to compare across subjects or conditions. Additionally, baseline-zero adjustment was performed to shift the starting point of the signal to zero, helping to clearly observe task-related changes over time. After filtering, the modified Beer-Lambert law was used to convert the optical signals into concentration changes of oxyhemoglobin (ΔHbO) and deoxyhemoglobin (ΔHbR) (Kocsis et al., 2006).

### 2.5 Data structure

The dataset is organized and distributed in the shared near infrared file format (.snirf), a standardized format designed to facilitate the sharing and analysis of fNIRS data. It comprises recordings from 22 participants, each performing six distinct motor imagery tasks. Correspondingly, the dataset is divided into six separate folders, each representing a specific activity (e.g., Left Ankle), with each folder containing data from all 22 participants for that particular task. Within these activity-specific folders, individual subject data are stored in separate subfolders named by participant ID. Each subfolder contains a pre-processed CSV file (e.g., sub1.csv) with filtered fNIRS signals, which can be used for classification and analysis tasks.

## 3 Summary

In summary, we present a structured and preprocessed fNIRS dataset focused on lower limb motor imagery tasks involving the ankle and knee joints. Collected from 22 participants, this dataset supports the development of real-time BCI applications for rehabilitation and assistive technologies. Its standardized format, clear organization, and emphasis on lower limb MI tasks make it a valuable resource for researchers aiming to advance decoding algorithms, improve motor function restoration strategies, and enhance the design of BCI-driven exoskeleton systems.

The study recruited limited number of participants, i.e., 22, though it is sufficient for initial testing, however the sample size may not fully capture the variability present in larger or more diverse populations.

## Data Availability

The original contributions presented in the study are publicly available. This data can be found here: https://figshare.com/s/c63ff90363263a4dd965; https://figshare.com/s/7ed1702717ae27dd3a03; https://figshare.com/s/307c1c7535a39b1cccfb; https://figshare.com/s/1577643d373cfde64399; https://figshare.com/s/fa8db647f18fe9696032; https://figshare.com/s/f99b3c84fbb148e87f7f.
